# *In Vitro* and *In Vivo* Evaluation of Autochthonous Probiotics and Their Effects on the Mucosal Health of Nile Tilapia (*Oreochromis niloticus*)

**DOI:** 10.3390/ani15223296

**Published:** 2025-11-15

**Authors:** Sherilyn T. Abarra, Sahya Maulu, Sheu G. Odu-Onikosi, Taofik A. Momoh, Benjamin Eynon, Matthew Emery, Mark Rawling, Daniel L. Merrifield

**Affiliations:** Fish Health and Nutrition Research Group, School of Biological and Marine Sciences, University of Plymouth, Plymouth PL4 8AA, UK; sahya.maulu@plymouth.ac.uk (S.M.); sheu.odu-onikosi@plymouth.ac.uk (S.G.O.-O.); taofik.momoh@plymouth.ac.uk (T.A.M.); ben.eynon@plymouth.ac.uk (B.E.); matthew.emery@plymouth.ac.uk (M.E.); mark.rawling@plymouth.ac.uk (M.R.)

**Keywords:** tilapia, autochthonous probiotics, *Bacillus subtilis*, goblet cells

## Abstract

The host microbiome is a promising source of probiotics for aquaculture species including Nile tilapia. In this study, the probiotic potential of autochthonous bacterial isolates from Nile tilapia and carp mid-intestines were screened *in vitro*. Two isolates (C61 and T70), closely related to *Bacillus subtilis*, exhibited antagonistic activity against multiple pathogen species and demonstrated multiple digestive enzyme activities. Their antagonistic activity in *Aeromonas hydrophila* assays remained even under simulated intestinal juice (SIJ) exposure. Subsequently, C61 (PT1) and T70 (PT2) were added to experimental diets at log 7 CFU/g of diet, and fed to Nile tilapia (5.32 ± 0.12 g) for 40 days. There were no significant differences observed in the growth performance across treatments. Despite limited *Bacillus* intestinal recovery levels, 16S rRNA gene metabarcoding revealed subtle shifts in the intestinal microbial community composition of the probiotic-fed groups. In addition, the PT1 group showed significantly longer mucosal fold length, elevated intestinal and skin goblet cell levels, and higher skin goblet cell coverage compared to the control. These results indicate the potential benefits of the isolates as functional feed additives for enhancing the mucosal health of Nile tilapia, but their benefits were likely achieved through transient activity given the low level of *Bacillus* recovery in the intestine.

## Simple Summary

Probiotics, which are beneficial microbes that support digestion and strengthen natural defences, offer a promising approach to support sustainable fish farming. In this study, bacteria naturally found in the gut of Nile tilapia were investigated to see if they could serve as probiotics. Two strains of bacteria were identified that could inhibit relevant fish pathogens and produce digestive enzymes. When these bacteria were added to the diet of the fish, it resulted in subtle shifts in intestinal microbial community composition. The probiotic-fed fish also displayed notable changes in the structure of their intestinal lining and in the production of mucus-secreting cells of the intestine and skin. These findings offer a positive contribution to tilapia probiotic research.

## 1. Introduction

Although plant-based proteins and lipids are commonly employed as substitutes for fishmeal and fish oil, their efficacy is often limited by the presence of antinutritional factors (ANFs), which can adversely affect nutrient digestibility and absorption [1,2]. One approach to addressing this challenge involves the incorporation of commercial enzymes into plant-based diets to enhance digestion, improve feed efficiency, and promote the growth of farmed fish. However, the application of commercial enzymes as feed additives has been limited by economic constraints, primarily driven by high production costs and elevated market prices [3]. Another significant concern associated with aquaculture intensification is the heightened susceptibility to disease, which is often exacerbated by increased stocking densities, poor feed digestibility, and deteriorating water quality [4]. Probiotics are widely used functional feed additives in aquaculture, with reported modes of action that can address problems such as poor nutrient digestibility and disease resistance. As such, the capacity to produce extracellular digestive enzymes and the ability to antagonise and inhibit pathogen colonisation are among the focal criteria in the selection of probiotics for aquaculture application.

Autochthonous probiotics, derived from aquatic environments, have attracted research attention to alleviate constraints on host- and strain-specific differences encountered in aquaculture applications [5]. Autochthonous probiotics populate the host’s mucus and epithelial surfaces [6] and are expected to be well-adapted in their natural environment in the gastrointestinal tract (GIT) of fish [7]. As an established natural defence system, the host microbiome is considered as a reservoir of potential probiotic strains, especially for cultured fish that are usually predisposed to antigenic stimuli and may have developed robust innate and adaptive immune responses against aquatic pathogens and stressors [7]. In a previous study, dietary administration of autochthonous *Bacillus* spp. (*B. velezensis*, *B. subtilis*, and *B. amyloliquefaciens*), which showed antagonism against *Streptococcus agalactiae in vitro*, reduced mortalities in Nile tilapia following *in vivo S. agalactiae* challenge (30–63% vs. 87% in controls) [8]. These findings demonstrate the benefits of probiotic treatment in enhancing disease resistance.

Previous research has explored the use of enzyme-producing probiotics, particularly those capable of secreting extracellular enzymes that degrade common ANFs [1,3]. Phytic acid is the principal storage form of phosphorus in plant-derived materials [9]. Due to its strong chelating properties, which bind essential mineral ions and reduce their bioavailability, it is considered an antinutrient [10]. Monogastric animals, including fish, lack endogenous intestinal phytase necessary for the digestion of phytic acid [11]. Previous studies [11,12,13] have isolated and characterised autochthonous phytase-producing bacteria from fish GIT as a potential strategy for dietary phytase supplementation; however, their *in vivo* use has rarely been explored.

The present study aimed to isolate and characterise autochthonous bacterial strains from the mid-intestine of *O. niloticus*, with a specific focus on evaluating their probiotic potential for safe application in aquaculture. Selected candidates demonstrating promising probiotic traits were further assessed for their antagonistic efficacy against *Aeromonas hydrophila* and phytate-degrading activity under simulated gastrointestinal conditions, to better understand their functional relevance in enhancing nutrient bioavailability. The two most promising strains were incorporated into experimental diets and orally administered to *O. niloticus* to evaluate their effects on growth performance, feed efficiency, and mucosal health. These findings are expected to inform the targeted development of host-specific probiotics for use in precision aquafeed formulations.

## 2. Materials and Methods

### 2.1. Bacterial Isolation and Culture

Autochthonous bacteria were isolated from the mid-intestine of *O. niloticus* (1.96 ± 0.60 g) as described elsewhere [14]. Intestinal bacteria were also isolated from mirror carp, *Cyprinus carpio* (2.54 ± 0.07 g), from a previous experiment following the same protocol. Intestinal samples (100 mg) were homogenised in 900 µL of phosphate-buffered saline (PBS; Merck Life Science UK Limited, Dorset, UK) and were subjected to serial dilution. For microbial culture, 100 µL of diluted homogenate was plated onto tryptic soy agar (TSA; Merck Life Science UK Limited, Dorset, UK) plates and incubated at 25 °C for 24 h. Distinct colonies were subsequently sub-cultured using the streak dilution method and used for *in vitro* screening.

### 2.2. In Vitro Assays

The autochthonous bacterial isolates were tested following the *in vitro* screening protocol outlined in Supplementary Figure S1.

#### 2.2.1. Pathogen Antagonism Assay

The pathogenic strains utilised in this study were obtained from the microbiology culture collection of the University of Plymouth. Detailed description of each pathogen with its respective culture conditions are provided in Supplementary Table S1.

Pathogen suspensions were prepared by suspending a single colony of each pathogen in sterile PBS and adjusting the optical density (OD_600_) to 0.1. Suspensions were evenly spread on agar plates with a sterile swab to create a uniform lawn. Probiotic candidates were cultured from single colonies in tryptic soy broth (TSB; Merck Life Science UK Limited, Dorset, UK) and incubated overnight at 25 °C (120 rpm). Wells (5 mm) were made in the agar using a sterile metal borer, and 25 µL of probiotic broth culture was pipetted into each well. Wells containing sterile TSB served as negative controls. Assay media and incubation conditions corresponded to the culture requirements of each pathogen (Supplementary Table S1). Plates were incubated for 24 h, after which zones of clearance were observed and recorded.

#### 2.2.2. Haemolytic Activity

Sheep blood agar plates were prepared by supplementing sterile TSA with 5% (*v*/*v*) defibrinated sheep blood (TCS Biosciences Ltd.; Buckingham, UK) at 47 °C. Each isolate was streaked onto the blood agar plates and incubated at 25 °C for 24 h. Haemolytic activity was assessed [15] as β-haemolysis (clear zones surrounding colonies), α-haemolysis (greenish discoloration around colonies), or γ-haemolysis (no observable change or clearing around colonies).

#### 2.2.3. Qualitative Screening for Extracellular Enzyme Activity

Probiotic candidate isolates were screened for phytase, xylanase, and tannase activity. To isolate and enumerate phytase-producing bacteria, a modified phytase-screening medium (MPSM) was used according to Roy et al. [12]. Bacterial isolates were streaked on the MPSM plates and were incubated at 30 °C for 7 d. The qualitative screening of phytase-producing bacterial isolates was performed [16] and zones of clearing around bacterial colonies were observed and recorded.

Bacterial isolates were subjected to qualitative screening for xylanase production [17]. To isolate xylanase-producing bacteria, isolates were streaked on xylan (XY) agar medium [18] and incubated at 30 °C for 24 h. Isolates cultured in XY plates were flooded with congo red solution (0.5% congo red; *w*/*v*) and 5% ethanol (*v*/*v*) for 5 min, followed by repeated decolorisation using 1 M of NaCl [19]. Positive xylanolytic activity was distinguished by the appearance of halo surrounding the bacterial colony against the red background.

To screen for tannase-producing bacteria, nutrient agar containing 1% (*w*/*v*) tannic acid solution was used [20]. Bacterial isolates were streaked on the nutrient agar plate and incubated at 37 °C for 48–72 h. The bacteria forming clear zones around the colonies were regarded to have extracellular tannase activity.

### 2.3. Probiotic Activity in Simulated Gastrointestinal Conditions

The best-performing probiotic candidate isolates from the initial *in vitro* assays (Phase 1; Supplementary Figure S1) were selected and further screened for their probiotic activity under simulated gastrointestinal conditions (Phase 2; Supplementary Figure S1). The assay was designed to simulate the gastric and intestinal pH of tilapia. Simulated gastric juice (SGJ, pH 1) and simulated intestinal juice (SIJ, pH 7) were prepared as described elsewhere [21].

#### 2.3.1. Pathogen Antagonism Against *A. hydrophila* in SIJ

*A. hydrophila* lawns and probiotic cultures (OD_600_ = 1, ~10^8^ CFU/mL) were prepared as in Section 2.2.1. Wells (4 mm) received (1) 20 µL of probiotic + 20 µL of NaCl, 0.5% *w*/*v*, (2) 20 µL of probiotic + 20 µL of filter-sterilised SIJ, (3) controls (20 µL of SIJ + 20 µL of NaCl, 0.5% *w*/*v*). Plates were incubated at 25 °C for 24 h, and inhibition zones were measured in triplicate [22,23].

#### 2.3.2. Phytate Degradation Activity in Simulated Gastrointestinal Fluids

Crude cell-free enzyme extracts were prepared and stored at −20 °C [24]. To assess phytate degradation under simulated gastrointestinal conditions, 200 µL of extract was pre-incubated with 200 µL of SGJ or SIJ at 37 °C for 60 min [25]. Phytase activity was determined using sodium phytate in acetate buffer (pH 5.0), and reactions stopped with 20% (*w*/*v*) trichloroacetic acid (TCA) solution; released phosphate quantified at 405 nm against a potassium monophosphate standard [26,27]. Phytase activity was calculated by applying the Beer–Lambert law:

A=ε×l×c

where A = absorbance;

ε = molar absorptivity constant (µM^−1^cm^−1^);
l = path length (1 cm);
c = concentration (µM).

One unit of phytase (U) was defined as the amount of enzyme that produced 1 µmol of inorganic phosphorous per min at 50 °C. To determine the specific activity, total protein concentration of the crude enzyme extract was determined by using Bradford micro assay [28] and adjusting the total assay volume to 2 mL of bovine serum albumin (100 µg/mL), which was used as the standard. Specific activity was defined as U per mg of protein.

### 2.4. Bacterial Isolate Sequencing and Identification

16S rRNA gene sequence analysis was used for bacterial identification of the probiotic candidate isolates as described elsewhere [29]. Bacterial genomic DNA was extracted using the QIAamp Fast DNA Stool Mini Kit (Qiagen Ltd.-UK; Manchester, UK) followed by amplification using universal primers 27F (5′-GAGTTTGATCATGGCTCAG-3′) and 1492R (5′-GGTTACCTTGTTACGACTT-3′). The PCR reaction mixture comprised 12.5 µL of PCRBIO Ultra Mix (PCR Biosystems Ltd., London, UK), 10.5 µL of diethylpyrocarbonate (DEPC)-treated water (Thermo Fisher Scientific Inc.; Cheshire, UK), 1 µL of each primer, and 1 µL of template DNA. Thermal cycling conditions consisted of an initial denaturation at 95 °C for 5 min, followed by 32 cycles at 94 °C for 15 s, 55 °C for 15 s, and 72 °C for 30 s. The final extension was at 72 °C for 1 min. Purified DNA samples were submitted to the DNA Sequencing and Services facility at the University of Dundee. Resulting sequences were analysed using the Basic Local Alignment Search Tool (BLAST; https://blast.ncbi.nlm.nih.gov (accessed on 13 December 2022)) to identify closely related species, with percent identities exceeding 98%. For phylogenetic analysis, chromatogram files (.ab1) were edited in MEGA (v.11.0.13) to generate a consensus sequence, which was exported in FASTA format. Sequence similarity was determined using BLASTn against the NCBI GenBank nucleotide database. Closely related strain sequences were retrieved from GenBank and aligned with the isolate sequence using ClustalW in MEGA. A neighbour-joining phylogenetic tree was constructed based on the Kimura 2-parameter model, and 1000 bootstrap replicates were used to evaluate the robustness of the tree’s topology.

### 2.5. Probiotic Diet Preparation

The basal diet was formulated using Animal Feed Formulation Software (AFOS Cloud 4.16) to meet the known nutrient requirements of Nile tilapia [30,31] (Table 1). Probiotic diets were prepared with isolates C61 (PT1) and T70 (PT2). Cultures were grown in TSB, incubated overnight at 25 °C (120 rpm), centrifuged (4000× *g*, 10 min), washed twice with PBS, and resuspended. Suspensions were top-sprayed onto the pelleted basal diet and homogenised; controls received sterile PBS. The diets were left to dry at room temperature for 24 h and were top-dressed with sunflower oil at 1% (*w/w*) while mixing to ensure uniform coating. The prepared diets were air-dried at room temperature for 24–48 h. Probiotic levels were 1.03 × 10^7^ CFU/g (PT1) and 1.67 × 10^7^ CFU/g (PT2), confirmed by spread-plating on TSA. Representative colonies were identified as *Bacillus subtilis* (Section 2.3). Diet composition was analysed per [32] (Table 2).

### 2.6. Experimental Design and Feeding

The growth trial was conducted in a recirculating aquaculture system (RAS) of the Tropical Unit Aquarium at the University of Plymouth. The RAS was equipped with a Kaldnes (K1) biofilter (Evolution Aqua Ltd.; Wigan, UK) in conjunction with heavy aeration from a separate air pump. The water exchange rate had approximately a four-times-per-hour turnover for each tub, delivered by a Swelluk Premium 5000 Pump (40 W; 5000 lph rating; Swell UK Ltd., Cheshire, UK). Water temperature (27.25 ± 0.05 °C), dissolved oxygen (7.33 ± 0.29 mg/L), and pH (6.73 ± 0.30) were monitored daily, while ammonium (0 mg/L), nitrite (0 mg/L) and nitrate (>10 mg/L) were monitored weekly; and all were maintained within the acceptable range for the fish species.

*O. niloticus* were procured from University of Stirling and transported to the University of Plymouth aquaria. After acclimation, Nile tilapia fingerlings (5.32 ± 0.12 g) were distributed randomly into experimental tanks with capacities of 30 L, filled up to 13 L of water with a stocking density of 22 fish per tank. Experimental diets were randomly assigned among nine tanks with three replicates per treatment. All fish were weighed prior to day one of feeding and feed intake was set at 3–4% of biomass per day. Daily feed was allocated into four equal rations and feed intake was adjusted every week by batch weighing after a 24 h feed deprivation period.

### 2.7. Growth Performance, Feed Utilisation, and Carcass Analyses

After the 40-day feeding trial, fish were euthanised following UK Home Office schedule 1 procedures [33]. Growth performance and feed utilisation were evaluated as final weight (FW), net wet gain (NWG), specific growth rate (SGR), feed intake (FI), feed conversion ratio (FCR), protein efficiency ratio (PER), condition factor (CF), and % survival (Supplementary Equations (S1)–(S6)). All calculations were based on each replicate tank treatment. Whole fish (three per replicate tank) were used for carcass analyses to calculate ash, protein, and fat/lipid content [32].

**Table 2:** Proximate composition of the experimental diets.

Component	Treatment		
	CON	PT1	PT2
Dry matter	93.2 ± 0.0	93.2 ± 0.1	93.2 ± 0.0
Protein *	43.5 ± 0.4	44.4 ± 0.4	44.1 ± 0.3
Lipid *	4.9 ± 0.1	5.1 ± 0.2	4.9 ± 0.2
Ash *	4.5 ± 0.3	4.7 ± 0.1	4.6 ± 0.2
NFE *^a^	47.1 ± 0.6	45.9 ± 0.6	46.4 ± 0.3
GE (kJ/g) ^b^	20.3 ± 0.0	20.4 ± 0.0	20.3 ± 0.1

* Wet weight basis. ^a^ NFE, nitrogen-free extract; ^b^ dietary gross energy (GE) was estimated from the analysed dietary macronutrient composition and the corresponding energy equivalents: 17.2 kJ g^−^^1^ for carbohydrates, 23.6 kJ g^−^^1^ for crude protein, and 39.5 kJ g^−^^1^ for lipids [34].

### 2.8. Histological and Electron Microscopy Analysis

For histological analysis, skin samples were obtained from the dorsal region, which is immediately above the lateral line and below the dorsal fin base, to ensure consistent sampling across individuals. Whole intestine was excised and the posterior segment (ca. 5 mm) of the intestine was collected. Skin and posterior intestine samples (n = 9 per treatment) were fixed in 10% formalin. Tissues were processed using a Leica TP1020 Auto Processor (Leica Microsystems Ltd.; Milton Keynes, UK) through graded IMS and embedded in paraffin; multiple consecutive tissues sections were cut at 4 µm (Leica RM2235, Leica Microsystems Ltd.; Milton Keynes, UK). Sections were mounted, dried overnight, and stained with Hematoxylin and Eosin (H&E) and Alcian blue van-Gieson (AB-vG). Sections were mounted with a coverslip and DPX and dried prior to imaging for morphometric analysis. The prepared slides were photographed using a Leica DMD 108 microimaging system (Leica Microsystems Ltd.; Milton Keynes, UK). H&E-stained samples were evaluated for mucosal fold length (MFL), lamina propria width (LPW), muscularis thickness (MT), and intraepithelial leukocyte (IEL) counts. ABvG-stained slides were analysed for goblet cell (GC) counts and coverage. Five mucosal folds were randomly selected for measurement from among those that were fully visible in the cross-section and representative of the overall intestinal morphology. Measurements were taken from folds distributed around the intestinal lumen to ensure that no single region of the section was overrepresented. Five intact mucosal folds per sample were used to measure MFL, LPW, IEL counts, and GC counts while MT per sample was determined from the mean of 10 measurements along random regions of the muscularis [35]. Image analysis was performed using the ImageJ 1.54d software (National Institutes of Health, Bethesda, MD, USA).

For scanning electron microscopy (SEM), SEM samples were rinsed with sodium cacodylate buffer (0.1 M, pH 7.2) and dehydrated using graded ethanol and hexamethyldisilazane (HMDS). Dried samples were mounted on aluminium stubs and gold-sputter-coated for SEM imaging. All electron micrographs were analysed with ImageJ 1.54d (National Institute of Health, USA) with ten representative micrographs analysed per sample [36].

### 2.9. Culture-Based Intestinal Microbiological Analysis

Each fish was dissected in aseptic conditions, and the mid-intestine (n = 9; 3 per replicate tank) was extracted for intestinal microbiological analysis. Mid-intestinal tissues including its contents (ca. 100 mg) were homogenised in 900 µL of PBS and subjected to 10-fold serial dilution up to 10^−^^6^. For total viable count (TVC) cultures, 100 µL of the diluted homogenate (10^−^^3^ to 10^−^^6^ dilutions) were spread onto the TSA plates and incubated at 25 °C for 24 h. For *Bacillus* agar cultures, 100 µL of the diluted homogenate (10^−^^2^ to 10^−^^4^ dilutions) were spread onto the *Bacillus* agar (ATCC Medium 455) [37] and incubated at 25 °C for 24 h. Based on colony morphology, colonies were counted from statistically viable plates to calculate CFU/g.

### 2.10. 16S rRNA Gene Metabarcoding Analysis

16S rRNA gene metabarcoding analysis was performed to examine the composition and structure of the microbial communities in the fish intestinal samples. Genomic DNA extractions from intestinal samples (n = 9; 3 per replicate tank) were performed using QIAamp PowerFecal Pro DNA kit (Qiagen Ltd.-UK; Manchester, UK), following the manufacturer’s protocol. Genomic DNA samples were sent to Novogene UK Company Ltd. (Cambridge, UK) for 16S rRNA gene metabarcoding of the V3–V4 region. Based on clean data, DADA2 was used to denoise and obtain initial ASVs (Amplicon Sequence Variants). Species annotation was performed using the QIIME2 software (QIIME2-202202) using Silva annotation database. Subsequent analysis of alpha diversity and beta diversity were all performed based on the output normalised data.

#### Diversity Indices

To analyse the diversity, richness, and uniformity of the microbial communities within the group, alpha diversity was calculated from Goods coverage, Chao1, Shannon, and Simpson indices in QIIME2. To evaluate the complexity of the community composition and compare the differences between groups, beta diversity was calculated based on weighted and unweighted unifrac distances in QIIME2. A cluster tree was constructed by the unweighted pair-group method with arithmetic mean (UPGMA), which is based on the weighted unifrac distance matrix.

### 2.11. Statistical Analyses

The collected data were checked for normality using Shapiro–Wilk’s test and Levene’s test of equality of variances was used to test for the homogeneity of variances. For growth performance, histology, and microbiology data with normal distribution, one-way ANOVA was used to calculate the levels of significant differences between more than two groups. Tukey post hoc test was conducted to compare between means at *p* ≤ 0.05. When the data violated the assumption of normality and homogeneity of variances, the Kruskal–Wallis test was conducted as an alternative to one-way ANOVA. For probiotic activity in simulated gastrointestinal conditions, the Mann–Whitney U test was used as a non-parametric test to compare means between two unrelated groups. Paired *T*-test was used to compare means of the same group at different time points. Significant difference was accepted at *p* ≤ 0.05. All data analysis was performed using SPSS v.28.0.1.1.

## 3. Results

### 3.1. In Vitro Assays

A total of 150 probiotic candidate isolates were successfully cultured and isolated from the intestinal contents of *C. carpio*, while 113 probiotic candidates were isolated from the *O. niloticus* intestines. These isolates were initially screened for antagonism against *A. hydrophila* and *Streptococcus iniae*.

The 31 best-performing isolates that demonstrated antagonism to both (*A. hydrophila* and *S. iniae*) or one of the pathogens were tested against three further fish pathogens (*Vibrio anguillarum*, *Vibrio parahaemolyticus*, and *Yersinia ruckeri*). These 31 isolates were also assessed for their haemolytic activity and extracellular enzyme (xylanase, phytase, and tannase) activity. The complete results of these assays for each of the 31 isolates are shown in Table 3.

### 3.2. 16S rRNA Gene Sequence Analysis

Table 3 shows the results of 16S bacterial rRNA gene sequencing with percent identities and accession numbers of closely related species for each probiotic candidate isolate. BLAST search results revealed that out of the 31 isolates, 27 were identified as *Bacillus* species (C16, C22, C24, C27, C29, C39, C54, C61, C80, C122, C123, C140, C141, C146, T30, T32, T56, T66, T67, T68, T70, T71, T72, T103, T105, T112, T113), one as *Pseudomonas mosselii* (C72), one as *Enterobacter* sp. (C150), one as *Plesiomonas shigelloides* (T65), and one as *Gottfriedia acidiceleris* (T69).

From the results of the initial *in vitro* assays (Phase 1, Supplementary Figure S1), isolates C61 and T70 were selected as the two best-performing isolates. These two isolates were α haemolytic, displayed antagonism to more pathogens, and exhibited extracellular enzyme activity to more enzymes than the other isolates.

**Figure 1:**
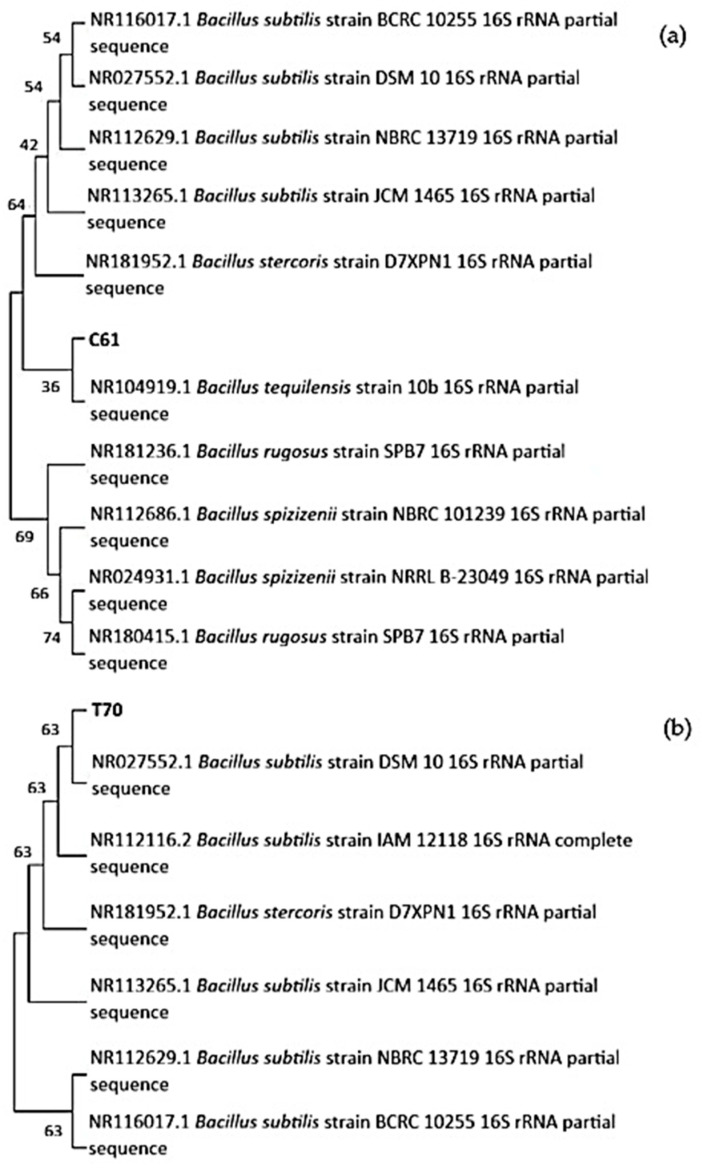
Neighbour-joining phylogenetic tree based on 16S rRNA gene sequences showing the relationship of isolate C61 (**a**)/T70 (**b**) and reference *Bacillus* species. The tree was constructed in MEGA using the Kimura 2-parameter model with 1000 bootstrap replicates. Bootstrap values are shown at branch nodes.

The 16S rRNA gene sequence of isolate C61 showed >99% similarity to both the *Bacillus tequilensis* strain 10b (NR104919.1) and *Bacillus subtilis* type strains in BLASTn analysis. In the neighbour-joining phylogenetic tree (Figure 1a), isolate C61 clustered with *B. tequilensis* (bootstrap value = 36) and grouped within the *Bacillus subtilis* species complex. The *B. subtilis* type strains formed a separate, moderately supported cluster (54–64%), while other *Bacillus* species such as *B. spizizenii* and *B. rugosus* formed distinct, well-supported lineages (>65%). Based on high sequence similarity and its phylogenetic position, isolate C61 was identified as a *Bacillus* sp. closely related to the *B. subtilis*–*B. tequilensis* group.

Isolate T70 showed >99% similarity to *Bacillus subtilis* type strains in BLASTn analysis. In the neighbour-joining phylogenetic tree (Figure 1b), isolate T70 clustered tightly with *B. subtilis* strains DSM 10, IAM 12118, JCM 1465, NBRC 13719, and BCRC 10255, with bootstrap support of 63%. The close grouping of T70 within the *B. subtilis* clade and its high sequence similarity indicate that the isolate is most likely a strain of *Bacillus subtilis* or a closely related member of the *B. subtilis* species complex.

The two isolates were subjected to further assays under simulated gastrointestinal conditions (Phase 2, Supplementary Figure S1).

### 3.3. Probiotic Activity in Simulated Gastrointestinal Conditions

#### 3.3.1. Pathogen Antagonism in SIJ

Isolates C61 and T70 were tested for pathogen antagonism against *A. hydrophila* under SIJ exposure. Table 4 shows the results of the assay for each probiotic candidate isolate. The degree of pathogen antagonistic activity of C61 with or without SIJ exposure was not significantly different to that of T70. The pathogen antagonism of C61 isolate did not significantly differ when exposed to SIJ. Meanwhile, the degree of pathogen antagonism of T70 was significantly reduced under SIJ exposure (*p* = 0.020).

#### 3.3.2. Phytate Degradation Activity in Simulated Gastrointestinal Fluids

**Figure 2:**
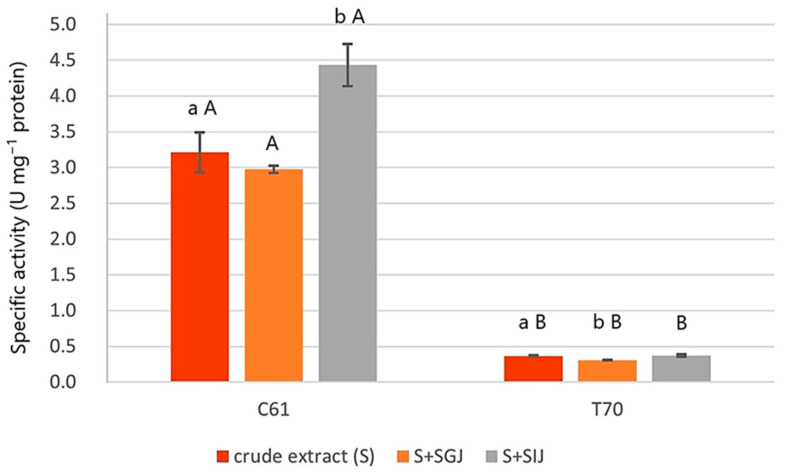
Phytase specific activity of C61 and T70 crude enzyme extract with and without SGJ and SIJ exposure. U mg^−1^ protein was defined as the amount of enzyme that produced 1 µmol of inorganic phosphorous per min at 50 °C per mg of protein. Data are expressed as mean ± SD (n = 3). Different lowercase letters on different bar colours denote significant difference; different uppercase letters on the same bar colours indicate significant difference (*p* ≤ 0.05).

Figure 2 shows the phytase-specific activity of the crude enzyme extracts from C61 and T70 probiotic candidate isolates with and without exposure to SGJ and SIJ. The specific activity of C61 crude extract (S) was significantly higher than T70 (*p* = 0.050). Moreover, C61 crude extract had a significantly higher specific activity than T70 under both SGJ (*p* = 0.043) and SIJ (*p* = 0.046) exposure. These results also reveal the effect of SGJ and SIJ exposure on the phytase-specific activity of the crude enzyme extract from each probiotic candidate isolate. C61crude extract (S) had a significantly higher specific activity with SIJ exposure (*p* = 0.008) than without, while the specific activity of T70 crude extract significantly decreased under SGJ exposure (*p* = 0.026).

### 3.4. Growth Performance, Feed Utilisation, and Carcass Composition

All experimental groups showed reasonable growth performance as revealed by the assessed growth parameters after the feeding trial (Table 5). There were no significant differences found between the treatment groups. Moreover, there were no significant differences observed in the fish carcass composition across all groups (Table 6).

### 3.5. Histological Analysis

**Figure 3:**
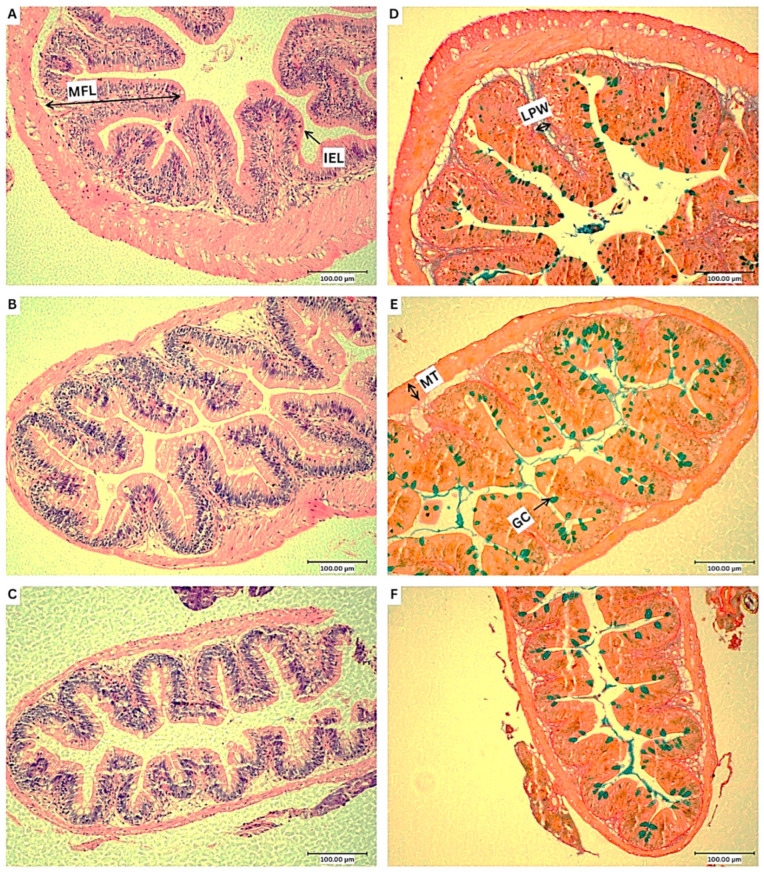
Representative photomicrographs from the histological analysis of the posterior intestine of *O. niloticus*-fed experimental diets. (**A**,**D**) control; (**B**,**E**) PT1; (**C**,**F**) PT2; H & E staining (**A**–**C**) and AB/vG staining (**D**–**F**). MFL, mucosal fold length; IEL, intraepithelial leukocyte; LPW, lamina propria width; MT, muscularis thickness; GC, goblet cell. Scale bars: 100 μm.

**Figure 4:**
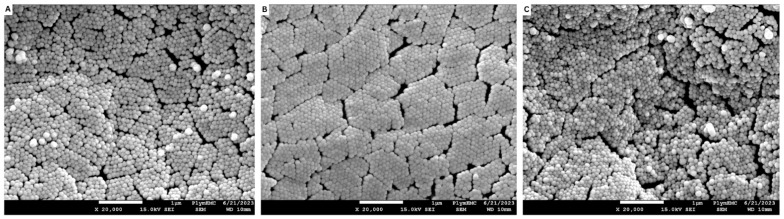
Representative electron micrographs from the posterior intestine of *O. niloticus*-fed experimental diets. (**A**) Control; (**B**) PT1; (**C**) PT2. Scale bars: 1 μm.

Histological analysis of the posterior intestine revealed intact epithelial lining with no signs of lesions, necrosis, or cellular detachment, indicating the absence of pathological damage in all treatment groups. Figure 3 shows the representative photomicrographs of the posterior intestine for each treatment group. Additionally, representative electron micrographs of the posterior intestine from SEM are presented in Figure 4. Table 7 presents the morphometrics data of the posterior intestine of Nile tilapia fed with experimental diets. The mucosal fold length of the PT1 group was significantly higher compared to the control (*p* = 0.006) and PT2 (*p* = 0.009)-fed groups. The muscularis thickness of the PT2 group was significantly lower than the control (*p* = 0.043) and PT1 (*p* = 0.020) groups. The goblet cell count was significantly higher in the PT1 group (*p* = 0.017) compared to the control regime.

**Figure 5:**
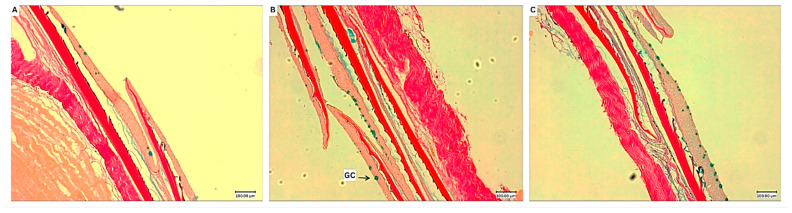
Representative photomicrographs from the skin histological analysis of *O. niloticus*-fed experimental diets. (**A**) Control; (**B**) PT1; (**C**) PT2. (**A**–**C**); AB/vG staining. GC, goblet cell. Scale bars: 100 μm.

**Figure 6:**
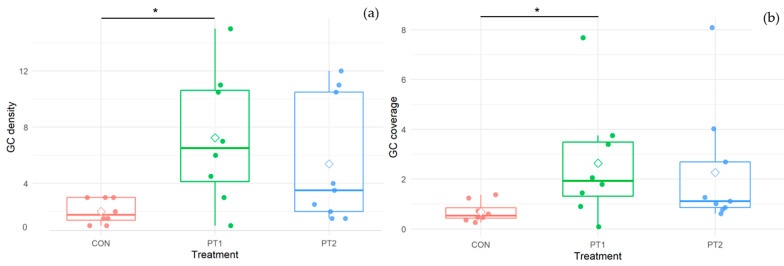
Skin goblet cell density (**a**) and coverage (%) (**b**) of *O. niloticus*-fed experimental diets. Boxes depict the interquartile range with the median indicated by the central line; whiskers extend to the most extreme data points, excluding outliers. Individual data points are plotted as dots, and the diamond represents the group mean. Asterisks (*) between columns indicate significant difference (*p* ≤ 0.05). GC, goblet cell.

Figure 5 shows the representative photomicrographs of skin histology for each treatment group. As presented in Figure 6, skin goblet cell density (*p* = 0.030) and coverage (*p* = 0.033) of the PT1 group were significantly higher compared to the control.

### 3.6. Intestinal Microbiological Analysis

The culture-based approach revealed no significant differences on the total viable counts of cultivable allochthonous bacteria present in the mid-intestine of fish across the treatments (Table 8). Presumptive *Bacillus* spp. levels were not significantly different across the groups.

### 3.7. Intestinal Microbiota Metabarcoding

**Figure 7:**
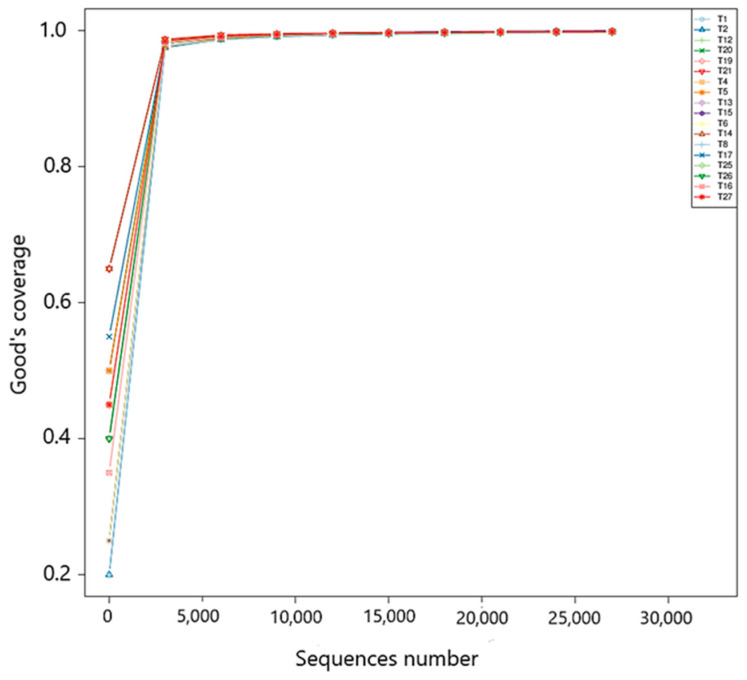
Good’s coverage rarefaction curve generated from sequences and ASVs obtained from each sample.

Out of 18 intestinal samples (n = 6 per treatment) from Nile tilapia fed with experimental diets, a total of 1,855,247 paired-end raw reads were obtained by 16S rRNA gene metabarcoding of the V3–V4 region. After data filtering to reveal clean tags, chimeric sequences were detected and removed to obtain 1,333,458 effective tags used for downstream analysis. The average number of effective tags from control group were 74,567, 72,198 from PT1 and 75,477 from PT2. After noise reduction using DADA2, ASVs were obtained, and Figure 7 presents the rarefaction curve of the Good’s coverage for each sample. The curve for each sample plateaued with all values close to one, indicating that the sequencing depth was sufficient to reflect the biodiversity of the samples.

**Figure 8:**
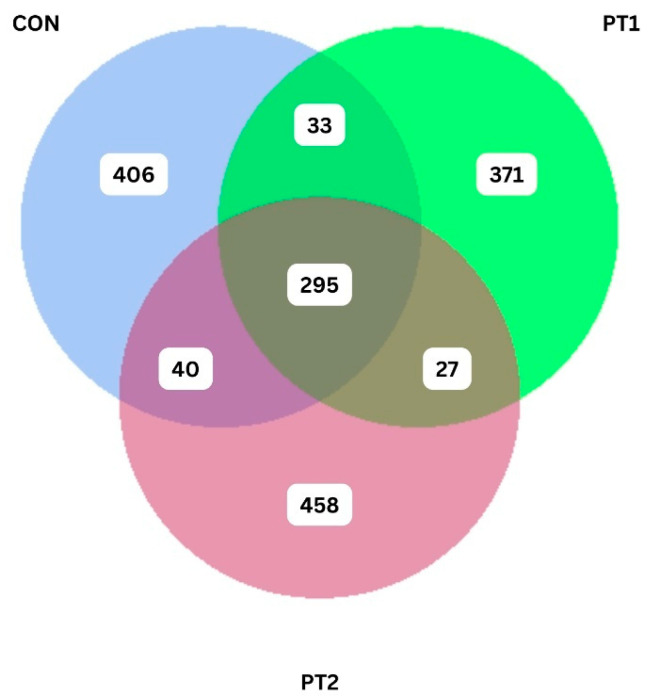
Unique and shared ASVs of the intestinal microbiota of Nile tilapia fed with experimental diets. CON, control; PT1, probiotic treatment 1; PT2, probiotic treatment 2.

Figure 8 shows the Venn diagram of the ASV generated from each treatment. The control group had a total of 406 unique ASVs, sharing 33 and 40 ASVs to PT1 and PT2 group, respectively. PT2 had 458 unique ASVs, higher than the control group and the PT1 group with only 371.

#### 3.7.1. Alpha Diversity Analysis

**Figure 9:**
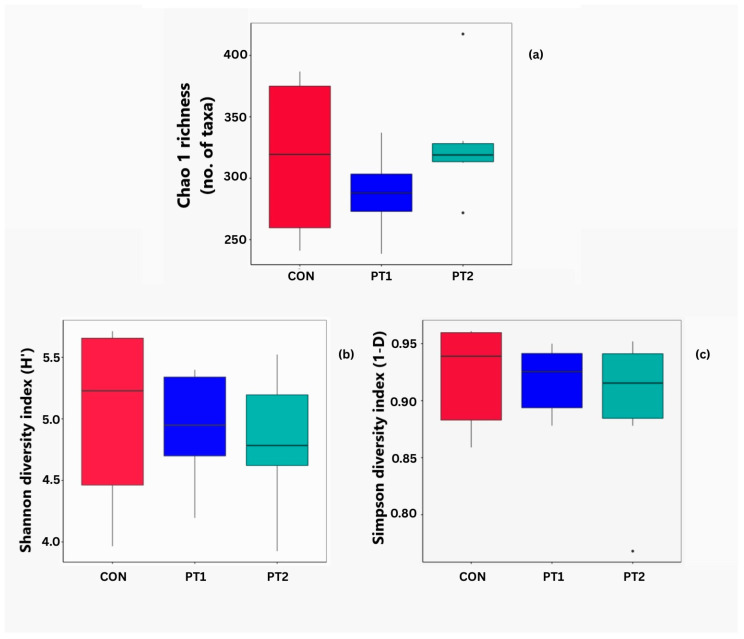
Alpha diversity of the intestinal microbiota shown in Chao1 (**a**), Shannon (**b**), and Simpson (**c**) indices. CON, control; PT1, probiotic treatment 1; PT2, probiotic treatment 2.

The alpha diversity indices of the intestinal microbiota of Nile tilapia fed with the experimental diets are shown in Figure 9. Chao1, Shannon, and Simpson indices were not significantly different across all groups (Figure 9a, Figure 9b, and Figure 9c, respectively).

#### 3.7.2. Beta Diversity Analysis

**Figure 10:**
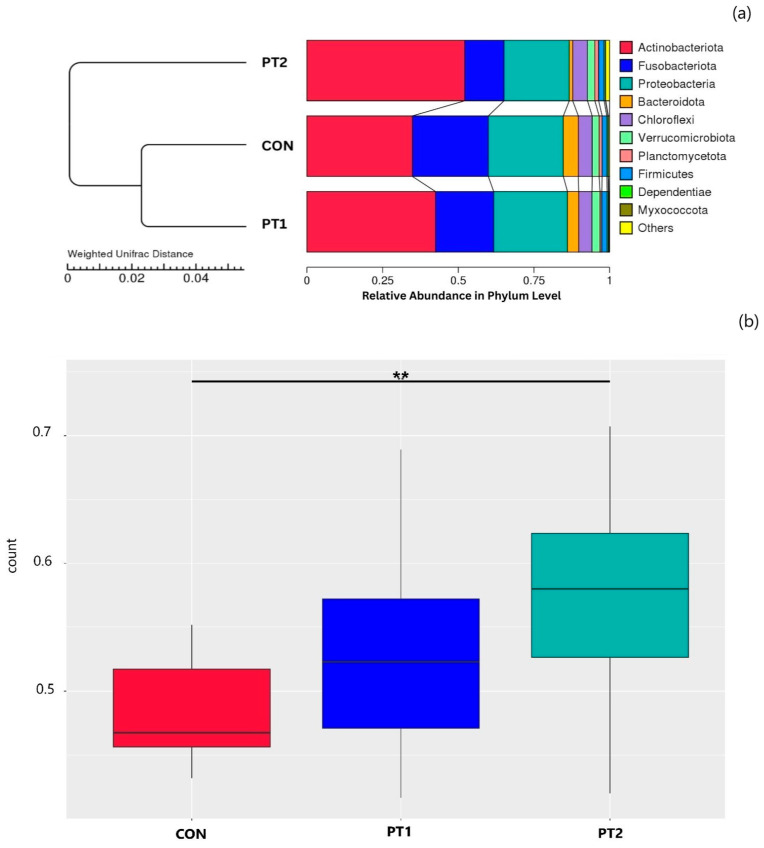
UPGMA cluster tree based on weighted Unifrac distance (**a**); beta diversity analysis based on unweighted UniFrac distance (**b**). Asterisks (**) between columns indicate significant difference (*p* ≤ 0.01). CON, control; PT1, probiotic treatment 1; PT2, probiotic treatment 2.

To describe variation in beta diversity, the dissimilarity coefficient between experimental groups was measured using weighted and unweighted UniFrac distances. To examine the similarity of the intestinal microbiota among the treatment groups, the unweighted pair-group method with arithmetic mean (UPGMA) method was used. As shown in the UPGMA cluster tree based on weighted UniFrac distance (Figure 10a), the control group was more similar to the PT1 group than the PT2 group. Based on the unweighted UniFrac distance beta diversity, there was a significant dissimilarity between the control and PT2 groups (Figure 10b).

**Figure 11:**
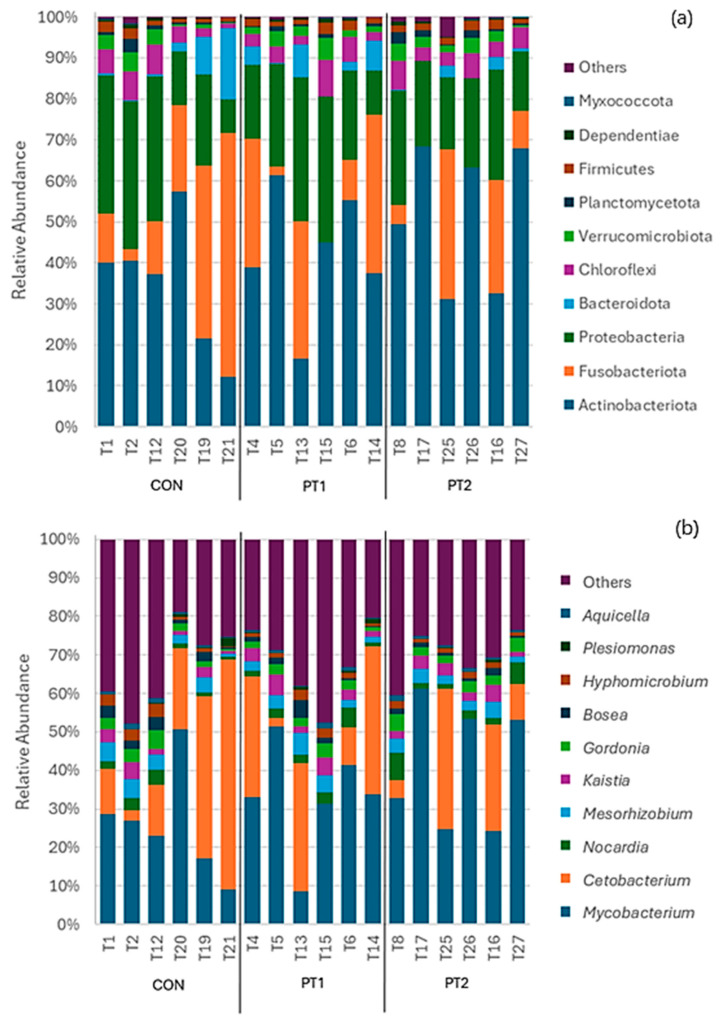

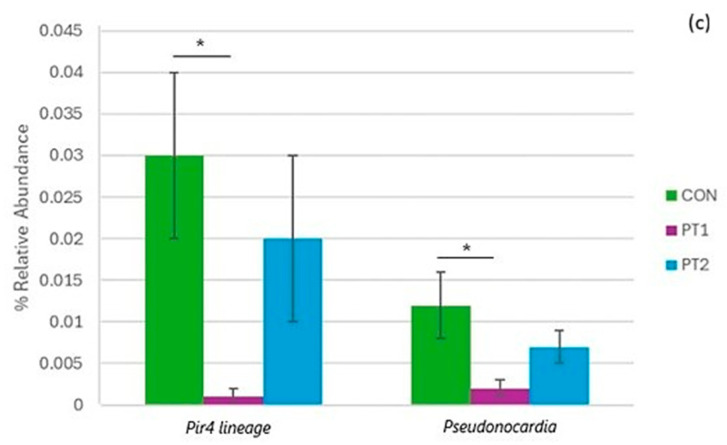
Relative abundance of the intestinal microbiota at phylum (**a**) and genus (**b**,**c**) levels. Values are expressed as mean ± standard error of the mean. Asterisks (*) between columns indicate significant difference (*p* ≤ 0.05). CON, control; PT1, probiotic treatment 1; PT2, probiotic treatment 2.

Figure 11 shows the relative abundance of the intestinal microbiota in different taxonomic levels. At the phylum level, the top 10 most abundant phyla were *Actinobacteria*, *Fusobacteriota*, *Proteobacteria*, *Bacteroidota*, *Chloroflexi*, *Verrucomicrobiota*, *Planctomycetota*, *Firmicutes*, *Dependentiae*, and *Myxococcota* (Figure 11a). At the genus level, the top 10 most abundant genera included *Mycobacterium*, *Cetobacterium*, *Nocardia*, *Mesorhizobium*, *Kaistia*, *Gordonia*, *Bosea*, *Hyphomicrobium*, *Plesiomonas*, and *Aquicella* (Figure 11b).

Further analysis of other relevant taxa at the genus level revealed significant variation between groups. Results showed that the relative abundance of *Pir4 lineage* in the control group was significantly higher compared to the PT1 group (*p* = 0.022). Moreover, *Pseudonocardia* relative abundance in the control was significantly higher compared to the PT1 group (*p* = 0.036) (Figure 11c). There were no significant differences between the treatment groups for the relative abundance of other taxa.

## 4. Discussion

### 4.1. In Vitro Screening

Disease susceptibility is often heightened in intensive fish farming systems, and this is further aggravated by environmental stressors [38]. This situation in fish farms is often exacerbated by pathogenic bacteria that commonly occur as opportunistic pathogens in already immunocompromised hosts [39]. This study conducted an *in vitro* screening for pathogen antagonism with the probiotic candidate isolates from Nile tilapia and mirror carp. The antagonistic activity of the isolates was tested against *A. hydrophila*, *S. iniae*, *V. anguillarum*, *V. parahaemolyticus*, and *Y. ruckeri* with the aim of isolating probiotic candidates for application in aquaculture. The results revealed that isolate C61 (closely related to *B. subtilis*–*B. tequilensis* group) can antagonise four out of the five pathogens tested. Seven more *Bacillus* sp. isolates (C27, C80, C122, C123, C140, C141, and T70) displayed antagonism against three out of the five pathogens tested. The pathogen-inhibitory intestinal bacterial community of freshwater teleosts was previously reported to be dominated by *Bacillus* species [40,41]. In previous reports, *Bacillus* spp. were found to inhibit pathogen colonisation via competitive exclusion and *B. subtilis*, in particular, can inhibit pathogens *in vivo* in both its vegetative form and as spores [42]. In line with the results of the present study, autochthonous *Bacillus* spp. isolated from the intestine of Nile tilapia [43], common carp [44], and other carp species [45,46] have been reported to exhibit antagonism against *A. hydrophila* and *V. parahaemolyticus* [47].

The genus *Aeromonas* contains several species that are pathogenic to freshwater fish and are considered as important pathogens in tilapia culture systems [38,39]. *A. hydrophila* is one of the most prevalent bacterial pathogens in tilapia culture and is reported to cause high mortalities in both wild and cultured fish [39]. Previous research on the infection route of *A. hydrophila* identified the gills and skin as the main portals of entry in freshwater fish species [48,49]. There are also studies that propose the fish intestine as an important infection route for *A. hydrophila* [49,50]. Therefore, dietary probiotics with capacity to antagonise pathogenic bacteria within the intestinal environment would be beneficial to enhance the protective barrier and prevent translocation of pathogenic bacteria across the intestinal barrier. The present study further evaluated the pathogen antagonistic activity of the two best-performing probiotic candidate isolates under SIJ. Isolate C61 retained 96% of its pathogen antagonism against *A. hydrophila* under SIJ exposure. For T70, the isolate retained 86% of its antagonistic activity under SIJ conditions. These results demonstrate the capacity of the two probiotic candidates to exhibit pathogen antagonistic activity in simulated intestinal conditions.

Apart from pathogen-inhibitory capacity, numerous members of the *Bacillus* genus in the fish intestinal microbiota were reported to produce digestive enzymes, including xylanase, tannase, and phytase as discussed in the review of Soltani et al. [51]. Comparable results were revealed in the current study evidenced by *Bacillus* spp. isolates exhibiting extracellular enzyme activity for two and all three enzymes tested. Out of the *Bacillus* spp. isolates that displayed extracellular enzyme activity for all three enzymes tested, five were closely related to *Bacillus subtilis* (C24, C123, C146, T67, and T70). In previous works, *B. subtilis* subsp. *spizizenii* isolated from the intestine of Indian major carp has demonstrated xylanase production [52], while tannase-producing *B. subtilis* was also isolated from Nile tilapia intestine [53].

Thermotolerance and broad pH stability are two of the most critical requirements for enzyme supplements in animal feed including phytases [54]. These two factors mainly determine the biochemical characteristics of the enzyme, their stability during feed processing and passage through the GIT of the animal [55]. In the current study, crude enzyme extracts from probiotic candidate isolates C61 and T70, both closely related to *B. subtilis*, were exposed to simulated gastrointestinal fluids. To the author’s knowledge, this is the first report of phytase-specific activity of *B. subtilis* strains under simulated gastrointestinal conditions. The phytase-specific activity of the crude enzyme extract from isolate C61 was at 3.21 U mg^−1^ protein, which was significantly higher than that of isolate T70 (0.37 U mg^−1^ protein). The specific activity from the crude enzyme of isolate C61 is comparable to the results of other previous studies on *B. subtilis* strains’ phytase-specific activity [56,57]. Under SIJ exposure, the specific activity of isolate C61 crude enzyme extract was significantly higher than without SIJ exposure. These findings demonstrate the potential of isolate C61 as a phytase-producing probiotic supplement in fish diets. As a strain of *B. subtilis* with an innate capacity to produce spores and withstand wide ranges of temperature and pH [42], isolate C61 may have the advantage of enhancing phytase activity in the anterior part of the intestine, which is the main site of nutrient absorption in fish.

### 4.2. In Vivo Trial

In the present study, the growth performance and feed utilisation of Nile tilapia fed with probiotic-supplemented diets were evaluated. After the 40-day feeding trial, there were no significant differences observed on the zootechnical parameters across all groups. This corresponds with a number of previous studies that investigated the effects of host-derived [58] Bacillus probiotic supplementation [59,60,61,62,63] on the growth performance of Nile tilapia. In contrast, dietary supplementation of host-derived *Bacillus* spp. (*B. velezensis*, *B. subtilis*, and *B. amyloliquefaciens*) at a dose of 1 × 10^8^ CFU/g in single and combined administration (1:1:1) resulted in significantly higher FW and weight gain (WG) and significantly lower FCR than the control diet-fed Nile tilapia after four weeks [8]. Intestinal autochthonous *B. megaterium* at a dose of 10^8^ CFU/g was supplemented in Nile tilapia diets for eight weeks. The probiotic-fed group exhibited significantly higher FW, WG, SGR, and FCR compared to the control group [5]. In another study using host-derived *B. subtilis* (1 × 10^6^ and 1 × 10^8^ CFU/g) diet supplementation, FW, WG, SGR, and FCR were significantly enhanced in Nile tilapia with fed probiotic diets compared to the control after 60 days [64]. These varied effects of dietary *Bacillus* supplementation on Nile tilapia growth performance revealed the multifaceted mechanism of these probiotics. A plethora of factors including the probiotic strain, dose, host species, life stage, system conditions, and experimental duration among others should be carefully considered when designing a growth trial.

In the current study, the total viable counts of presumptive *Bacillus* spp. in Nile tilapia mid-intestine were examined after the feeding trial via culture-based techniques and there were no significant differences found across groups. This is consistent with the results from 16S rRNA gene metabarcoding analysis of the intestinal microbiota with no significant changes on the relative abundance of *Bacillus* genus across treatments (i.e., control, 0.58%; PT1, 0.59%; PT2, 0.77%). This is similar to the findings of an earlier study where Nile tilapia diets were supplemented with *Bacillus velezensis* MT9 (10^6^ CFU/g) for 90 days. The results showed that *Bacillus* remained at low relative abundance in the intestine, ranging from 0.06 to 0.14% in the control group and 0.01 to 0.31% in the probiotic-fed group [65]. Another study did not observe significant differences in the total intestinal *Bacillus* CFU counts between the control (6.41 log CFU/g) and the treatment group (5.86 log CFU/g) after a 28-day feeding trial with *Bacillus subtilis* probiotic supplementation (1.34 × 10^7^ CFU/g) [66]. In relation to the present study, the low level of *Bacillus* spp. detected in the intestine across experimental groups may reflect that the intestinal microbiome under these conditions appears to have a competitively limiting or possibly even excluding effect. This suggests that even when adding additional populations, the *Bacillus* population does not appear to be able to expand to form a larger proportion of the community. This may also be evident in some wild Nile tilapia populations where *Bacillus*, despite being identified as a member of the core microbiota, were detected at relatively low abundance (0.01–0.3%) in the midgut region [67]. In an earlier study [68], Calsporin^®^, a *B. subtilis* probiotic (10^10^ CFU/g) product, was used as feed additive in koi carp; however, *B. subtilis* was not detected in the intestine of any of the groups using 16S rDNA-V3 PCR-DGGE after 35 days of feeding. Nonetheless, the probiotic-fed group exhibited significant improvement on growth performance (FW, WG, FCR) compared to the control, which warrants further investigation whether intestinal colonisation is a requirement for probiotics to elicit beneficial effects to the host. More importantly, future studies should focus on the impact of probiotic persistence in the gastrointestinal tract, which may be primarily influenced by their metabolic activities and mode of action.

The results of the 16S rRNA gene metabarcoding of the intestinal microbiota revealed that probiotic supplementation did not significantly affect the total number of ASVs or alpha diversity indices, indicating that overall species richness and within-sample diversity remained stable across treatments. Moreover, the relative abundance of dominant phyla and genera did not change significantly, suggesting that core microbial communities were maintained across groups. Previous studies have reported the impacts of dietary probiotics on the alpha and beta diversity of the intestinal microbiota of Nile tilapia [69,70]. On the contrary, a meta-analysis evaluation reported no differentiation patterns in the alpha and beta diversity metrics in tilapia gut microbiota as an effect of dietary probiotics and other feed additives [71]. In the current study, beta diversity analysis (unweighted UniFrac) showed a significant dissimilarity between the PT2 and control groups, suggesting that the presence/absence of certain microbial lineages was altered in response to probiotic inclusion. Consistently, the UPGMA tree based on weighted UniFrac distances indicated that the control and PT1 groups clustered closely together, while PT2 formed a distinct branch, reflecting greater divergence and compositional shifts in both the identity and abundance of taxa. These results are in line with the previous findings [72] on the effects of host-associated *Rummeliibacillus* sp. and *Microbacterium* sp. dietary supplementation (1 × 10^8^ CFU/g) in the intestinal microbial community of olive flounder. Beta diversity reported using principal coordinate analysis (PCoA) of unweighted Unifrac metrics revealed notable differences between the intestinal microbiota of probiotic and control groups [72]. Beta diversity metrics estimate the community distance or similarity across samples [72] and were reported to be more sensitive than alpha diversity indices in observing differences in microbial community composition [73].

Metabarcoding analysis of the intestinal microbiota of Nile tilapia after the feeding trial revealed the most abundant phyla across all groups. This includes *Actinobacteria*, *Fusobacteriota*, *Proteobacteriota*, *Bacteroidota*, *Verrumicrobiota* and *Firmicutes*, which were also reported previously as the most common phyla in the intestinal microbiome of cichlids and tilapia [69,74,75]. *Actinobacteria* were reported to produce metabolites for aquatic pathogen inhibition [76], while *Firmicutes* abundance revealed negative correlation with pathogenic bacterial population in the intestinal surfaces [77]. An examination of the intestinal microbiota in farmed Nile tilapia revealed *Proteobacteria*, *Fusobacteriota*, and *Firmicutes* to be among the predominantly abundant phyla with predicted KEGG functions on carbohydrate and amino acid metabolism as well as signal transduction [78]. Moreover, phyla *Bacteroidota* and *Verrumicrobiota* were previously suggested to facilitate polysaccharide hydrolysis, carbohydrate fermentation, and short chain fatty acid production that can enhance intestinal barrier integrity [79,80].

In the present study, the genus *Cetobacterium* was reported as the second most relatively abundant genus in the intestinal microbial community of Nile tilapia juvenile fed with experimental diets. Previous studies have revealed that *Cetobacterium* belongs to the core microbial communities of tilapia intestines and are among the most dominant taxa [81,82,83]. *Cetobacterium somerae* strains were previously reported to produce Vitamin B12 [84]. In the current study, the relative abundance of genus *Plesiomonas* was detected at 0.20–0.46% in the intestinal microbiota of Nile tilapia juveniles fed with experimental diets. Members of the *Plesiomonas* genus are considered as potential pathogens in freshwater fish but were also detected as predominant in the microbiome in tilapia [3,85]. Other potentially pathogenic genera were detected across groups such as *Mesorhizobium*, *Nocardia*, *Gordonia*, and *Kaistia*. Martínez–Lara et al. [86] first reported the association of *Gordonia* in granulomatosis in fish. Some species of *Mycobacterium*, such as *Mycobacterium marinum*, cause mycobacteriosis and significant mortality in tilapia [87,88]. However, as with other diseases, *Mycobacterium* pathology depends on the species and susceptibility of the host [89]. *Mycobacterium* was one of the most abundant genera across all groups (26–42%) in the current study and this is consistent with previous reports [81,82,83]. Giatsis et al. [90] studied the impact of rearing environment (active suspension and recirculating aquaculture systems) on the gut microbiota of tilapia larvae and found *Mycobacterium llatzerense* present at high relative abundance (12–19%) in all gut samples. In another study on adult *O. mossambicus*, *Mycobacterium* was detected at above 25% relative abundance in the intestine of tilapia collected from a natural lake, suggesting its persistence in the gut during later growth stages in specific environments [91]. Importantly, in the current study, *Mycobacterium* relative abundance did not differ significantly among treatments, indicating that probiotic supplementation did not increase or suppress this group. No clinical signs of mycobacteriosis (e.g., skin lesions, nodules, emaciation) were observed in any fish in the present study.

In less common taxa, the relative abundance of genus *Pir4 lineage* was revealed to be significantly higher in the control compared to the PT1-fed group. *Pir4 lineage* is an uncultured member of family *Pirellulaceae*; hence, its characterisation is largely unknown. *Pir4 lineage* is closely related to genus *Pirellula* and thrive in low-oxygen habitats such as in soil [92], as well as in freshwater [93] and marine environments [94]. In Nile tilapia, significantly higher relative abundance of *Pirellula* was observed in the intestine of fish fed with high plant-based diet [95], as well as in shrimp biofloc supplemented with non-starch polysaccharide (NSP) [96]. Another less common taxa, *Pseudonocardia*, displayed statistically higher relative abundance in the intestine of the control-fed group compared to PT1. As ubiquitous soil bacteria, some species of *Pseudonocardia* can establish a symbiotic relationship with fungus-farming leaf-cutter ants [97] and have been an important subject for bioprospecting due to their anti-fungal properties [98]. Given the limited *Bacillus* recovery levels in the PT1-fed group, the significant reduction in these less common genera may reflect either a direct response to the transient metabolic or antagonistic activity of the *Bacillus* strains (e.g., production of inhibitory metabolites, nutrient competition, or localised changes in gut conditions) or an indirect, secondary response to shifts in other microbial taxa activities/metabolites or host-mediated effects. This suggests that even minimal, undetectable, or nonviable populations of *Bacillus* cells or spores within the fish gastrointestinal tract may still exert measurable influences on the host microbiota [65,99].

Probiotics can influence the histology of the intestine by altering tissue morphology, organisation, and cell differentiation in the intestinal lining [100]. Previous works on *Bacillus* spp. probiotics have documented its modulation of intestinal morphology in Nile tilapia [5,100,101,102]. In the current study, the mucosal fold length of the PT1-fed group was significantly longer than the control and PT2 group. Meanwhile, the intestinal goblet cell counts of the PT1-fed group was significantly higher than the control. These results were in line with previous reports where host-derived *Bacillus* probiotic (*Bacillus velezensis*, *B. subtilis*, and *Bacillus amyloliquefaciens*; 1 × 10^8^ CFU/mL) supplementation in Nile tilapia diets resulted in significant increase in mucosal fold height and goblet cell counts after four weeks of feeding [74]. In another study, significantly higher mucosal fold length was observed in Nile tilapia fed with autochthonous *B. subtilis*-supplemented diet (1 × 10^8^ CFU/g) for eight weeks [101]. These findings indicate that PT1 treatment can enhance the absorptive surface area of Nile tilapia intestine by increasing the mucosal fold length and mucus production from goblet cells.

Fish are in constant exposure to its aquatic environment and mucosal surfaces act as the first line of defence against external assault and potentially invading pathogens. The skin and intestinal epithelia represent key components of the mucosa-associated lymphoid tissue (MALT), which forms an integral part of the fish mucosal immune system [99]. In the current research, PT1 group exhibited significantly higher intestinal goblet cell counts and skin goblet cell coverage and density compared to the control group. Goblet cells secrete mucus that act as a protective overlay and a transport medium between the lumen and epithelial cells [103]. Commensal microbiota in the intestinal surfaces rely on mucus and undigested dietary carbohydrates for energy source and binding sites, developing a symbiotic interaction with the host [104]. Probiotics can enhance the production of mucus by stimulating goblet cells, thereby strengthening the mucosal barrier, protecting the gut from pathogens, and supporting overall intestinal health [100]. In line with the results of this study, *Bacillus* spp. probiotic supplementation in tilapia diets were reported to significantly elevate the number of intestinal goblet cells, implying a higher capacity to produce mucus [64,74,105,106,107,108]. Other previous research on probiotics revealed its influence on intestinal goblet cell counts and density [109,110,111,112]. The intestinal microbiota may influence goblet cell activities and modulate mucus production either by releasing bioactive compounds locally, or by triggering immune cells of the systemic immunity [113]. There is increasing evidence of the intestinal microbiota as regulators of the gut–skin axis with a balanced gut microbiome contributing to skin homeostasis [114], although there are limited published reports on the effects of dietary probiotics on fish skin goblet cell counts. A previous study on *Poecilopsis gracilis* demonstrated significant increase in skin mucus protein after feeding with a probiotic-enriched diet (*Artemia nauplii* enriched with *Lactobacillus casei*, 0.7 × 10^8^ CFU/mL) for 11 weeks compared to the control [115]. Increased protein content is indicative of enhanced mucus production, suggesting the probiotic effect of *L. casei* on skin mucosal immune defence [116]. In tilapia aquaculture, feed additives capable of stimulating goblet cell proliferation and mucus secretion in the skin could be highly desirable, since they can strengthen the first line of defence against pathogens, like *A. hydrophila* that utilises the skin as a primary infection route.

The effects of PT1 treatment on the intestinal and skin histology of Nile tilapia after the feeding trial were particularly noteworthy, especially since both culture-based and molecular analyses showed limited recovery of *Bacillus* in the intestine. It is also worth mentioning that PT1 treatment displayed significant increase in intestinal goblet counts but not coverage compared to the control-fed group, implying that there were higher number of goblet cells with smaller cell size. This may suggest the subtle effects of the probiotic treatment in increasing goblet cell turnover while maintaining mucosal homeostasis. With limited *Bacillus* recovery in the intestine, it is possible that the probiotic elicited a transient effect during gut transit. Such outcomes are consistent with the recognised transient-action mechanisms of *Bacillus* probiotics, whereby functional activity can modulate community composition and host physiology without stable colonisation [65,68]. A postbiotic effect may also be a plausible mechanism of action supported by previous studies on inactivated bacterial cellular components and their capacity to stimulate goblet cell dynamics and mucus production at mucosal sites [113,116,117]. These results are consistent with transient or postbiotic activity, as proposed for other *Bacillus* probiotics; and future studies focused on inactivated/postbiotic components (e.g., peptidoglycan, endospores, etc.) are necessary to better understand their mechanistic effects.

It is also worth noting that PT2 treatment resulted in significantly reduced muscularis thickness of the posterior intestine compared to the control and PT1 groups. Whilst the nominal doses of both probiotics in the diet was log 7 CFU g^−1^, the actual dose was marginally different, with PT2 containing 0.21 log_10_ units higher CFU g^−1^. This minor difference might have contributed to the different outcomes observed for each probiotic to some degree. The muscularis is composed of circular and longitudinal smooth muscle layers that determine intestinal motility [118]. Decrease in muscularis thickness may reduce peristaltic tone and mechanical strain for the intestine, leading to lesser contractile effort [119]. Reduced peristaltic activity may prolong luminal residence time, providing more ecological niches and temporal opportunities for microbial diversification. This may be the potential explanation for the significant dissimilarity in the beta diversity of microbial communities in the mid-intestine of PT2 compared to the control. An interactive host–microbiota adaptation may also explain the feedback effect of microbial restructuring on gut morphology.

## 5. Conclusions

In summary, the majority of the cultivable autochthonous probiotic candidates isolated from the intestines of Nile tilapia and mirror carp were members of the *Bacillus* genus and have demonstrated pathogen antagonism and extracellular enzyme activity *in vitro*. The two best-performing *B. subtilis* strains were subjected to further *in vitro* assays and exhibited promising probiotic properties (*A. hydrophila* antagonism and phytase-specific activity) under simulated gastrointestinal conditions. These findings highlight the potential of the probiotic candidates for enhancing nutrient digestibility and disease resistance. Dietary administration of both candidate probiotics did not result in any adverse effect on growth and intestinal health, which highlights their safe application. Despite the lack of growth benefits, both probiotic candidates influenced the intestinal microbial diversity and elicited subtle shifts in microbial community composition. PT1 treatment demonstrated benefits by significantly increasing mucosal fold length and goblet cell counts in the intestine, as well as the skin goblet cell density and coverage of Nile tilapia. Future research should assess how dietary supplementation with these candidate probiotics influences the immune fate of mucosal tissues, including the gut, gill, and skin, and evaluate functional outcomes such as pathogen resistance and immune cell activity to clarify their immunomodulatory potential. Furthermore, future studies should focus on growth optimisation and disease resistance validation to determine the more suitable probiotic candidate for industrial application.

## Figures and Tables

**Table 1:** Diet formulation of experimental diets.

Ingredient (g/100 g of Diet)	Treatment		
	CON	PT1	PT2
PT1 concentration *	--	7	--
PT2 concentration *	--	--	7
Soybean meal ^a^	38.0	38.0	38.0
Sunflower meal ^b^	25.0	25.0	25.0
Corn gluten meal ^a^	21.7	21.7	21.7
Cornstarch	8.7	8.7	8.7
Sunflower oil	3.2	3.2	3.2
Fish meal ^c^	1.0	1.0	1.0
Fish oil	0.5	0.5	0.5
Lysine HCl	0.5	0.5	0.5
Vitamin and mineral premix ^d^	0.5	0.5	0.5
CMC-binder	0.5	0.5	0.5
Gelatin	0.5	0.5	0.5

* Probiotic concentration is expressed as log CFU/g. Values are expressed as mean ± standard deviation (SD). ^a^ SKT. ^b^ Biomar. ^c^ Coppens. ^d^ Premier nutrition vitamin/mineral premix (contains 121 g kg^−1^ of calcium, 5.2 g kg^−1^ of phosphorous, 15.6 g kg^−1^ of magnesium, 250 mg kg^−1^ of copper (as cupric sulphate), 7.0 g kg^−1^ of Vit E (as alpha-tocopherol acetate), 1.0 μg kg^−1^ of Vit A, 0.1 μg kg^−1^ of Vit D3, and 787 g kg^−1^ of ash).

**Table 3:** Identification and characterisation of 31 autochthonous probiotic candidates isolated from mirror carp and Nile tilapia.

Isolate No.	Related Species	Identity (%)	Accession	Pathogen Antagonism					Haemolytic Activity	Extracellular Enzyme Activity		
				AH	SI	VA	VP	YR		Xylanase	Phytase	Tannase
C16	uncultured *Bacilli* bacterium	98.75%	MH375377.1	+	+	−	−	−	β	+	+	−
C22	*Bacillus* sp.	99.89%	OL679725.1; OL679711.1	+	+	−	−	−	α	+	+	−
C24	*Bacillus* sp.	99.89%	MT427735.1 OL679725.1; OL679711.1	+	+	−	−	−	β	+	+	+
C27	*Bacillus subtilis*	100.00%	MZ352777.1	+	+	+	−	−	α	+	+	−
C29	*Bacillus* sp.	100.00%	OL679725.1; OL679711.1	+	+	−	−	−	α	+	+	+
C39	*Bacillus subtilis*	99.51%	KF535143.1; GU193980.1	+	+	−	−	−	α	+	+	−
C54	*Bacillus subtilis*	87.85%	FR849706.1	+	+	−	−	−	α	+	−	−
C61	*Bacillus tequilensis and Bacillus subtilis strains*	>99%	NR104919.1	+	+	+	−	+	α	+	+	−
C72	*Pseudomonas mosselii*	99.72%	MT598025.1	+	−	+	+	+	no growth	+	+	+
C80	*Bacillus subtilis*	99.83%	OP904234.1	+	+	+	−	−	β	+	+	−
C122	*Bacillus subtilis*	99.07%	MT538257.1	+	+	+	−	−	β	+	+	−
C123	*Bacillus subtilis*	99.30%	KX426654.1; KX426653.1	+	+	+	−	−	β	+	+	+
C140	*Bacillus* sp.	99.89%	OL679725.1; OL679711.1	+	+	+	−	−	β	+	+	−
C141	*Bacillus velezensis*	99.91%	OP060623.1	+	+	+	−	−	β	+	+	−
C146	*Bacillus subtilis*	99.90%	CP026662.1	+	+	−	−	−	β	+	+	+
C150	*Enterobacter* sp. 18A13	99.82%	AP019634.1	+	−	−	−	−	γ	+	+	+
T30	*Bacillus stercoris*	99.76%	MN704462.1	+	−	−	−	−	β	+	+	+
T32	*Bacillus subtilis*	96.86%	MN631028.1	+	−	−	−	−	β	+	−	−
T56	*Bacillus subtilis* subsp. *subtilis*	96.47%	CP032855.1	+	+	−	−	−	β	+	+	−
T65	*Plesiomonas shigelloides*	94.15%	CP050969.1; LT575468.1; KU517709.1	−	+	−	−	−	γ	no growth	no growth	no growth
T66	*Bacillus subtilis*	98.43%	OM980686.1	−	+	−	−	−	α	+	+	−
T67	*Bacillus subtilis*	95.73%	MN894000.1	+	+	−	−	−	β	+	+	+
T68	*Bacillus subtilis*	100.00%	OP942174.1	+	+	−	−	−	α	+	+	−
T69	*Gottfriedia acidiceleris*	99.66%	MF101038.1	−	+	−	−	−	no growth	−	−	−
T70	*Bacillus subtilis*	99.70%	NR027552.1	+	+	+	−	−	α	+	+	+
T71	*Bacillus thuringiensis*	99.56%	KX822158.1	−	+	−	−	−	β	no growth	no growth	no growth
T72	*Bacillus subtilis*	98.06%	KX426661.1	−	+	−	−	−	α	+	+	−
T103	*Bacillus* sp.	99.35%	OL679725.1; OL679711.1	−	+	−	−	−	α	+	+	−
T105	*Bacillus tequilensis*	99.49%	MK296524.1	−	+	−	−	−	α	+	+	+
T112	*Bacillus subtilis*	95.42%	ON243943.1	+	−	−	−	−	α	+	+	−
T113	*Bacillus tequilensis*	95.73%	JX979116.1	+	+	-	-	-	α	+	+	-

AH, *A. hydrophila*; SI, *S. iniae*; VA, *V. anguillarum*; VP, *V. parahaemolyticus*; YR, *Y. ruckeri*; + (inhibition/positive); − (non inhibition/negative); α, alpha haemolysis; β, beta haemolysis; γ, gamma haemolysis.

**Table 4:** Pathogen antagonism of isolates C61 and T70 against *A. hydrophila* with and without SIJ exposure.

Isolate	Zone of Inhibition (mm)	
	Without SIJ Exposure	With SIJ Exposure
C61	15.33 ± 0.58	14.67 ± 0.58
T70	16.33 ± 0.58 ^a^	14.00 ± 1.00 ^b^

Values are expressed as mean ± SD (n = 3). Superscript letters in the same row indicate significant difference (*p* ≤ 0.05).

**Table 5:** Growth performance of Nile tilapia fed with probiotic-supplemented diets.

Parameter	Treatment		
	CON	PT1	PT2
IW (g fish^−1^)	5.4 ± 0.0	5.2 ± 0.2	5.4 ± 0.0
FW (g fish^−1^)	11.8 ± 0.5	11.8 ± 0.5	12.5 ± 0.6
NWG (g fish^−1^)	6.4 ± 0.5	6.5 ± 0.4	7.1 ± 0.6
SGR (% day^−1^)	1.9 ± 0.1	1.9 ± 0.1	2.0 ± 0.1
FI (g fish^−1^)	9.3 ± 0.4	9.5 ± 1.0	9.7 ± 0.4
FCR (g g^−1^)	1.5 ± 0.1	1.5 ± 0.1	1.4 ± 0.1
PER	1.5 ± 0.1	1.5 ± 0.1	1.6 ± 0.1
CF	1.7 ± 0.1	1.7 ± 0.0	1.7 ± 0.0
% Survival	98.5 ± 2.6	95.5 ± 7.9	97.0 ± 5.3

Values are expressed as mean ± SD. CON, control; PT1, probiotic treatment 1; PT2, probiotic treatment 2; IW, initial weight; FW, final weight; NWG, net weight gain; SGR, specific growth rate; FI, feed intake; FCR, feed conversion ratio; PER, protein efficiency ratio; CF, condition factor.

**Table 6:** Carcass composition of Nile tilapia fed with probiotic-supplemented diets.

Component (%)	Treatment		
	CON	PT1	PT2
Moisture	73.6 ± 0.9	74.4 ± 1.2	74.4 ± 0.8
Protein	57.4 ± 0.9	58.0 ± 3.1	59.0 ± 0.5
Lipid	25.9 ± 2.7	24.9 ± 3.4	23.6 ± 1.1
Ash	10.6 ± 0.8	10.4 ± 0.9	10.6 ± 0.4

% Moisture is reported on a wet weight basis, while % protein, % lipid, and % ash are expressed on a dry matter basis. Values are expressed as mean ± SD. CON, control; PT1, probiotic treatment 1; PT2, probiotic treatment 2.

**Table 7:** Posterior intestine morphometrics of Nile tilapia fed with probiotic-supplemented diets.

Parameter	Treatment		
	CON	PT1	PT2
Mucosal fold length (µm)	120.6 ± 29.5 ^a^	166.0 ± 27.5 ^b^	123.0 ± 27.1 ^a^
Lamina propia width (µm)	22.1 ± 5.6	21.0 ± 8.2	18.3 ± 4.0
Muscularis thickness (µm)	21.7 ± 11.3 ^a^	22.5 ± 9.5 ^a^	11.3 ± 3.3 ^b^
Goblet cell count (n/70 µm)	5.6 ± 2.6 ^a^	9.3 ± 2.4 ^b^	8.1 ± 2.8 ^ab^
% Goblet cell coverage (n/70 µm)	5.5 ± 4.5	6.8 ± 3.4	6.7 ± 2.8
IEL count (n/70 µm)	25.8 ± 2.7	29.5 ± 11.1	32.6 ± 10.1
Microvilli density (AU)	30.1 ± 6.3	24.1 ± 12.3	27.7 ± 11.7

Values are expressed as mean ± SD (n = 9). Different letters in the same row indicate significant difference (*p* ≤ 0.05). CON, control; PT1, probiotic treatment 1; PT2, probiotic treatment 2; IELs, intraepithelial leukocytes.

**Table 8:** Total viable counts (log CFU/g) of total cultivable allochthonous and presumptive *Bacillus* spp. in the mid-intestine of Nile tilapia fed with probiotic-supplemented diets.

	Treatment		
	CON	PT1	PT2
allochthonous	7.0 ± 0.4	6.9 ± 0.4	7.1 ± 0.2
*Bacillus* spp.	5.4 ± 0.3	5.5 ± 0.4	4.9 ± 0.7

Values are expressed as mean ± SD (n = 9). CON, control; PT1, probiotic treatment 1; PT2, probiotic treatment 2.

## Data Availability

The 16S rRNA gene sequencing data generated in this study have been deposited and are openly available in the NCBI Sequence Read Archive (SRA) under the accession number PRJNA1336401 and are accessible via the following link: https://www.ncbi.nlm.nih.gov/sra/PRJNA1336401 (accessed on 9 October 2025). All other data are available upon request from corresponding authors.

## Supplementary Materials

The following supporting information can be downloaded at https://www.mdpi.com/article/10.3390/ani15223296/s1; Figure S1: *In vitro* screening protocol in selecting the probiotic candidate isolates from mirror carp and Nile tilapia; Table S1: Selected pathogens and culture conditions; Equation (S1): net weight gain; Equation (S2): specific growth rate; Equation (S3): feed conversion ratio; Equation (S4): protein efficiency ratio; Equation (S5): condition factor; Equation (S6): % survival.
