# Supplementary material for: In Vitro and In Vivo Evaluation of Autochthonous Probiotics and Their Effects on the Mucosal Health of Nile Tilapia (Oreochromis niloticus)
Source: Animals (Basel). 2025 Nov 15;15(22):3296. doi: 10.3390/ani15223296 (PMC12649282; doi:10.3390/ani15223296)
Supplement: Supplementary file 1 [file animals-15-03296-s001.zip › Supplementary Materials.pdf]

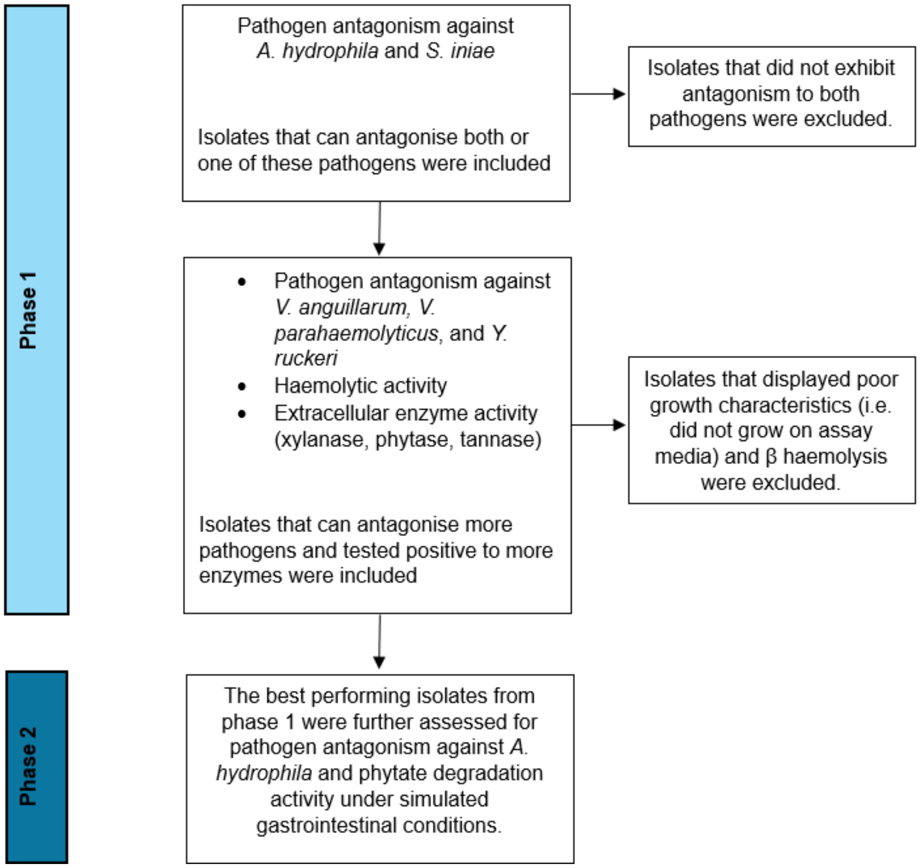

Figure S1. *In vitro* screening protocol in selecting the probiotic candidate isolates from mirror carp and Nile tilapia

Table S1. Selected pathogens and culture conditions.

Pathogen	Culture Media	Incubation Temperature
Aeromonas hydrophila[a]	TSA	25°C
Streptococcus iniae[b]	TSA	37°C (in CO_2 incubator)
Vibrio anguillarum[c]	TSA (with 2% NaCl)	26°C
Vibrio parahaemolyticus[d]	TSA	26°C
Yersinia ruckeri[e]	TSA	26°C

Bacterial strain codes or source/description: a, NCIMB 1134; b, NCIMB 702722; c, NCIMB 572; d, CM86 - Isolate from PHS Truro (Now UKHPA); e, 4/85 – Isolate from MAFF (Now DEFRA)

Equation S1

$$NWG = \text{mean } FW\ (g) - \text{mean } IW\ (g)$$

Equation S2

$SGR = (ln(FW) - ln(IW))\ x\ 100/T$; where: T = no. of days of the feeding trial

Equation S3

$FCR = FI/WG$; where: WG = weight gain (g)

Equation S4

$PER = WG/PI$; where: PI = protein intake (g)

Equation S5

$CF = (FW/(Lf)3)\ x\ 100$; where: Lf = fork length (cm)

Equation S6

$\%\ survival = F2/F1\ x\ 100$; where: F2 = no. of fish at the end of the trial, F1 = no. fish at the start of the trial
